# Craniectomy size and decompression of the temporal base using the altered posterior question-mark incision for decompressive hemicraniectomy

**DOI:** 10.1038/s41598-023-37689-7

**Published:** 2023-07-14

**Authors:** A. Früh, A. Zdunczyk, S. Wolf, R. Mertens, P. Spindler, D. Wasilewski, N. Hecht, S. Bayerl, J. Onken, L. Wessels, K. Faust, P. Vajkoczy, P. Truckenmueller

**Affiliations:** 1 Department of Neurosurgery, Charité-Universitätsmedizin Berlin, Corporate Member of Freie Universität Berlin, and Humboldt-Universität zu Berlin, and Berlin Institute of Health, Charitéplatz 1, 10117 Berlin, Germany; 2 Berlin Institute of Health, BIH Academy, Junior Digital Scientist Program, Charité-Universitätsmedizin Berlin, Berlin, Germany; 3 Berlin Institute of Health, BIH Academy, Junior Clinician Scientist Program, Charité-Universitätsmedizin Berlin, Berlin, Germany

**Keywords:** Neuroscience, Neurology

## Abstract

The altered posterior question-mark incision for decompressive hemicraniectomy (DHC) was proposed to reduce the risk of intraoperative injury of the superficial temporal artery (STA) and demonstrated a reduced rate of wound-healing disorders after cranioplasty. However, decompression size during DHC is essential and it remains unclear if the new incision type allows for an equally effective decompression. Therefore, this study evaluated the efficacy of the altered posterior question-mark incision for craniectomy size and decompression of the temporal base and assessed intraoperative complications compared to a modified standard reversed question-mark incision. The authors retrospectively identified 69 patients who underwent DHC from 2019 to 2022. Decompression and preservation of the STA was assessed on postoperative CT scans and CT or MR angiography. Forty-two patients underwent DHC with the standard reversed and 27 patients with the altered posterior question-mark incision. The distance of the margin of the craniectomy to the temporal base was 6.9 mm in the modified standard reversed and 7.2 mm in the altered posterior question-mark group (p = 0.77). There was no difference between the craniectomy sizes of 158.8 mm and 158.2 mm, respectively (p = 0.45), and there was no difference in the rate of accidental opening of the mastoid air cells. In both groups, no transverse/sigmoid sinus was injured. Twenty-four out of 42 patients in the modified standard and 22/27 patients in the altered posterior question-mark group had a postoperative angiography, and the STA was preserved in all cases in both groups. Twelve (29%) and 5 (19%) patients underwent revision due to wound-healing disorders after DHC, respectively (p = 0.34). There was no difference in duration of surgery. Thus, the altered posterior question-mark incision demonstrated technically equivalent and allows for an equally effective craniectomy size and decompression of the temporal base without increasing risks of intraoperative complications. Previously described reduction in wound-healing complications and cranioplasty failures needs to be confirmed in prospective studies to demonstrate the superiority of the altered posterior question-mark incision.

## Introduction

Decompressive hemicraniectomy (DHC) is common practice for various life-threatening indications^1–5^. However, this straightforward procedure still constitutes a particular challenging and, in some points, controversial surgery that exposes the patients to significant risks for various intraoperative and postoperative complications^6–9^. Many of the DHC-associated complications are related to the significant skin and bone flap compared to most standard supratentorial craniotomies. Wound-healing problems are one of the most frequently encountered complications after DHC and the main reason for the higher rate of wound-healing disorders is supposed to be due to injuring the superficial temporal artery (STA), which constitutes the main blood supply of the large myocutaneous flap^6,8,10^. This might happen, since the universally used standard reversed question-mark incision for DHC starts anterior to the tragus, where the STA runs superficially over the temporal root of the zygoma and is jeopardized during incision^8,11^. Injuring the STA during the skin incision predisposes the wound to ischemia and consecutive wound-healing disorders^12^. Therefore, the altered posterior question-mark incision starting behind the ear and posterior of the base of the mastoid process was proposed^13,14^. This incision type is supposed to preserve the STA and demonstrated a significantly reduced rate of infections and wound-healing disorders after cranioplasty^11^. However, it still remains unclear if this incision allows for the exact same size of the bone flap and for a sufficient exploration and decompression of the temporal base compared to the standard reversed question-mark incision. Further, it has been suggested that there is an increased risk of accidentally opening the mastoid air cells or injuring the transverse and sigmoid sinuses^12,15^. This might happen if the skin flap behind the ear is retracted too vigorously and if the craniotomy crosses below the superior nuchal line. Even the external auditory canal might be damaged^16^. Therefore, in this study, we compared the size of the craniectomy and the decompression of the temporal base using the altered posterior question-mark incision and our modified standard reversed question-mark incision. This modified reversed question-mark incision starts superior to the pinna to preserve the STA but allows for an adequate retraction of the scalp flap. We further analyzed intraoperative complications associated with the posterior approach and assessed the rate of infections and wound-healing disorders after DHC.

## Methods

### Patient cohort

This is a retrospective single center study performed at our tertiary medical center. Ethical approval was granted by *the ethics committee of the Charité–Universitätsmedizin Berlin* (EA1/012/22), who also confirmed waiver of informed patients consent based on the retrospective nature of our study. Data acquisition and presentation were made according to the STROBE (Strengthening the Reporting of Observational Studies in Epidemiology) guidelines for reporting observational studies^17^. All methods were performed in accordance with the Declaration of Helsinki.

We included all consecutive patients over the age of 18 years who underwent decompressive hemicraniectomy for a malignant hemispheric stroke, traumatic brain injury or intracerebral hemorrhage from 2019 to 2022. Patients that died before receiving the first postoperative CT were excluded. Demographic, clinical and radiographic patient data were retrospectively extracted from clinical records and documentation. They included patient-specific risk factors for wound-healing disorders (body-mass-index [BMI], diabetes mellitus type I and II [DM I/II], smoking, hypertension, chronic kidney disease, coronary or peripheral artery disease and previous strokes).

### Incision techniques and procedure

**Figure 1:**
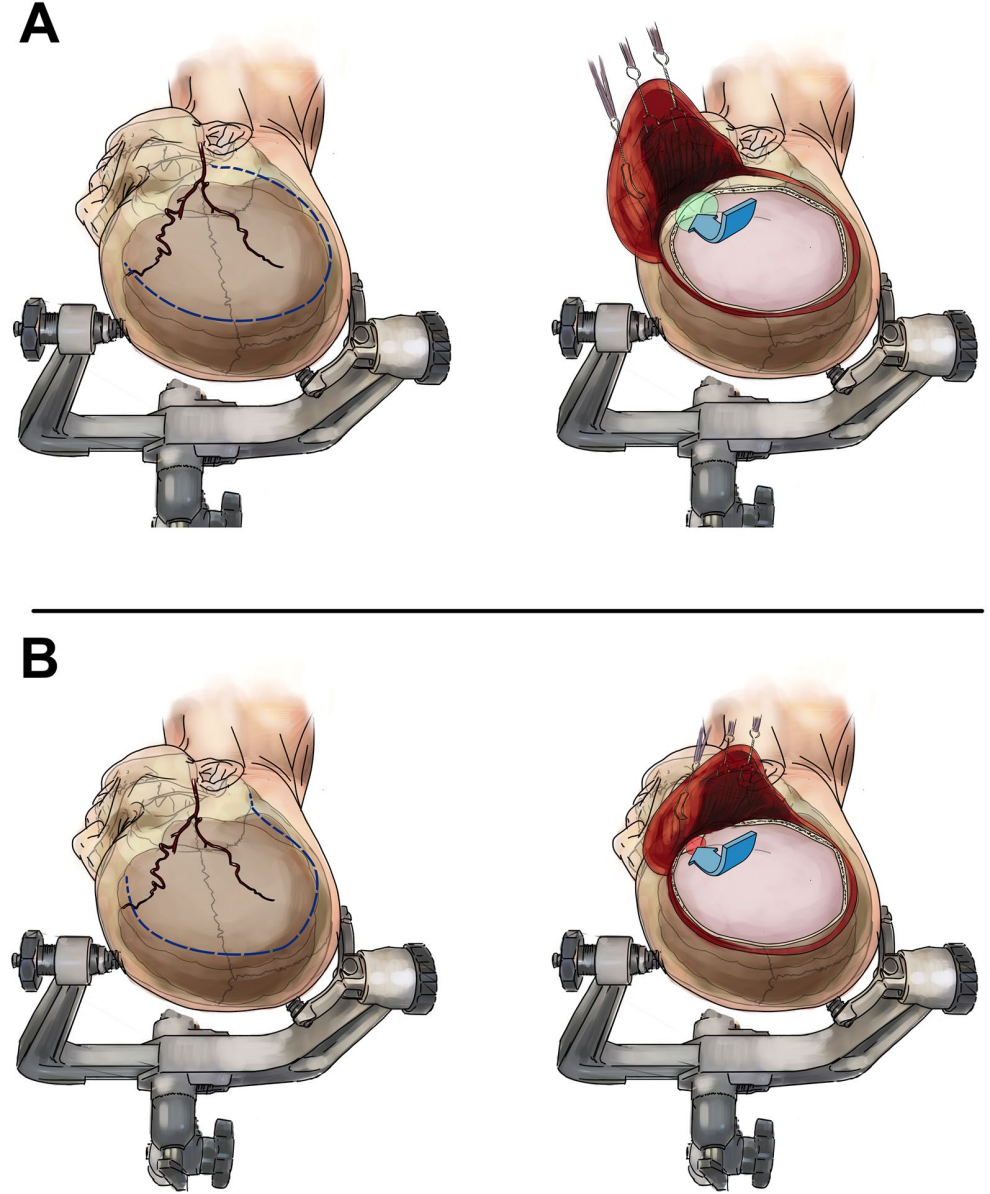
Incision techniques and corresponding exposure of the temporal bone. (A) Modified standard reverse question-mark incision with allegedly better access to the temporal base, (B) altered posterior question-mark incision with supposedly difficult exploration of the temporal base due to impeded reflection of the myocutaneous flap anterioinferiorely.

DHC was performed as previously described^18–20^. Before January 2021 we performed the standard reverse question-mark incision for DHC. However, we modified the incision and started the incision line superior to the pinna without extending down to the zygomatic arch. Starting superior and slightly anterior to the ear allows for an effective retraction of the scalp flap anteroinferiorly without jeopardizing the STA (Fig. 1). After the publication of the reduced infection rates after cranioplasty due to the altered posterior question-mark incision, we changed our clinical standard and adapted the proposed altered posterior question-mark incision^11,13^. Patients were divided into two groups according to the incision type that has been used: (a) treated via the modified standard reverse question-mark incision, (b) altered posterior question-mark incision (Fig. 1). According to our clinical routine, patients received a single shot of Cefazolin (2 g) and mannitol (50 mg/kg). In patients with known allergy to Cefazolin, 600-900 mg of intravenous Clindamycin was substituted. Aimed minimum size of the hemicraniectomy was 12 × 15 cm. After durotomy, the dura was replaced loosely over the cortex. Intraoperatively, each patient received a parenchymal intracranial pressure (ICP) probe and/or external ventricular drainage (EVD). Postoperative all patients were referred to a neurointensive care unit and were treated according to the current guidelines of German Society of Neurosurgery. In detail, within the first 24 h after surgery, systolic blood pressure (SBP) < 140 mmHg, mean cerebral perfusion pressure (CPP) > 65 mmHg, normothermia < 37.5 °C and normoglycemia were maintained. A routine postoperative CT scan was performed within 24 h after surgery.

### Outcomes

**Figure 2:**
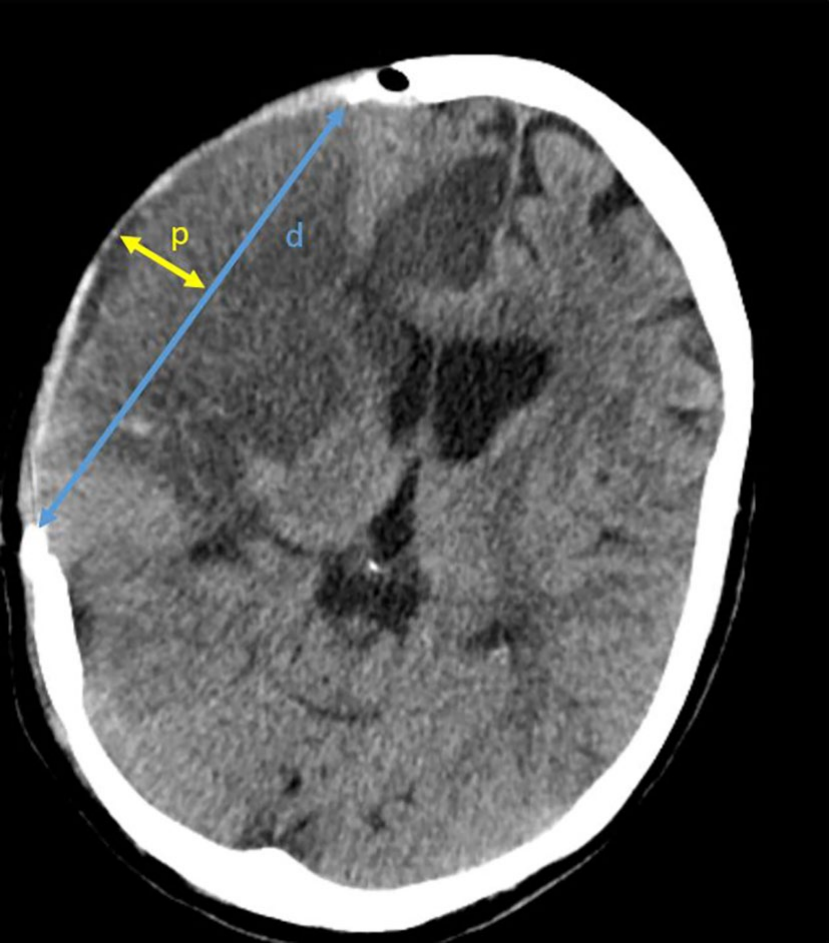
External brain herniation and decompression size assessed on postoperative CT scan. d was defined as the largest diameter of the craniectomy and p as the perpendicular line with the longest distance from the diameter to the dural flap at the same section.

Postoperative CT scans were analyzed independently by two neurosurgeons that were unaware if the modified standard reversed or the altered posterior question-mark incision was performed. Further, to assess the risk of external brain herniation depending on the craniectomy size, CT scan parameters were determined according to Münch et al. (Fig. 2)^21^. If a postoperative CT or MRI angiography was available, it was determined if the STA was preserved during surgery.

### Questionnaire

Personal experience of the surgeons with the altered posterior question-mark incision was evaluated with questionnaires on a four-point scale (1: excellent, 2: good but not optimal, 3: poor but acceptable, 4: unacceptable). The following issues were addressed: Decompression of temporal base, exploration of and access to the temporal base, occipital decompression, control of bleedings, intraoperative localization of the ear as landmark for further steps, protection of the ear, size of craniectomy, feasibility of wound closure. The questionnaire was distributed after the surgeon performed the altered posterior question-mark incision for the first time.

### Statistics

Statistical analyses were performed with GraphPad Prism 8.4.2. and SPSS version 25 (IBM Corp). Discrete data were presented as counts and percentage and compared by Chi-Square-Test. Continuous data were presented as mean and standard deviation (SD) and analyzed by Mann–Whitney-U-Test.

## Results

### Patient cohort

A total of 69 patients with postoperative imaging after DHC were included. 42 patients underwent DHC using the modified standard reverse question-mark incision and 27 using the altered posterior question-mark incision. The mean age of the study population was 51 ± 12 years. Major underlying pathologies were malignant hemispheric stroke (62.3%) and severe traumatic brain injury (23.2%). The baseline characteristic of the study population is provided in Table 1.

**Table 1:** Baseline characteristics of the study population and stratification regarding incision technique.

	Total study population (n = 69)	RQM (n = 42)	RA (n = 27)	p-value
Age, yr, mean $\pm$ SD	51 $\pm$ 12	50 $\pm$ 14	54 $\pm$ 10	n.s. (p = 0.51)
Female sex, n (%)	41 (59.4)	32 (76.2)	9 (33.3)	**sig (p < 0.001)**
BMI, kg/m^2^, mean $\pm$ SD	26.2 $\pm$ 4.2	25.7 $\pm$ 4.5	26.9 $\pm$ 3.9	n.s. (p = 0.307)
DM II, n (%)	11 (17.4)	5 (11.9)	7 (25.9)	n.s. (p = 0.134)
Smoking, n (%)	12 (16.4)	7 (16.7)	5 (18.5)	n.s. (p = 0.843)
Hypertension, n (%)	32 (46.4)	18 (42.9)	14 (51.9)	n.s. (p = 0.465)
NI, n (%)	4 (5.8)	2 (4.8)	2 (7.4)	n.s. (p = 0.646)
CAD, n (%)	10 (14.5)	8 (19.0)	2 (7.4)	n.s. (p = 0.180)
Prior stroke, n (%)	6 (8.7)	2 (4.8)	4 (14.8)	n.s. (p = 0.148)
Reason for DHC (%)				n.s. (p = 0.493)
MHS, n (%)	43 (62.3)	28 (66.7)	15 (55.6)	
TBI, n (%)	16 (23.2)	10 (23.8)	6 (22.2)	
SAH, n (%)	3 (4.3)	1 (2.4)	2 (7.4)	
ICH, n (%)	5 (7.2)	2 (4.8)	4 (14.8)	
Other, n (%)	1 (1.4)	1 (2.4)	0 (0.0)	
Postoperative lumbar drainage, n (%)	10 (14.5)	8 (19.0)	2 (7.4)	n.s. (p = 0.180)

BMI: Body mass index, CAD: Chronic arterial disease, DHC: decompressive hemicraniectomy, DM: Diabetes mellitus, IQR: interquartile range, n: number, CAD/PADP: Chronic arterial disease, MHS: malignant hemispheric stroke NI: Kidney insufficiency, RQM: standard reversed question-mark incision, RA: altered posterior question-mark incision, yr: years.

Significant values are in bold.

### Decompression

Both groups showed similar outcomes concerning the extent of decompression (Table 2). The distance of the lower margin of the craniectomy to the temporal base after decompressive hemicraniectomy was similar between the two incision groups, with 6.9 mm in the modified standard reversed question-mark group and 7.2 mm in the altered posterior question-mark group (p = 0.768). Further, the maximum diameter of the craniectomy showed no difference with 158.8 mm vs. 158.2 mm, respectively (p = 0.453). The state of the basal cisterns, which were open primarily, were similar in both groups (p = 0.228) and there was no increased sign for herniation on the postoperative CT scans. The ratio d/p, that represents the potential expansion volume of the decompressed brain, and the midline shift showed no difference between both groups.

**Table 2:** Performance of decompression.

	Total study population (n = 69)	RQM (n = 42)	RA (n = 27)	p-value
First postoperative CT scan				
Rest to temporal base, mm, mean $\pm$ SD	7.1 $\pm$ 5.2	6.9 $\pm$ 5.4	7.2 $\pm$ 4.9	n.s. (0.768)
Max Diameter, mm, mean $\pm$ SD	158.6 $\pm$ 9.8	158.8 $\pm$ 8.8	158.2 $\pm$ 11.5	n.s. (0.453)
d/p ratio, median, mean $\pm$ SD	3.0 $\pm$ 0.4	3.1 $\pm$ 0.4	3.0 $\pm$ 0.4	n.s. (0.392)
Midline shift, mm, mean $\pm$ SD	4.4 $\pm$ 4.0	4.3 $\pm$ 3.9	4.5 $\pm$ 4.1	n.s. (0.941)
Signs of herniation				n.s. (0.238)
None	64 (92.8)	40 (95.2)	24 (88.9)	
Subfalcine	4 (5.8)	1 (2.4)	3 (11.1)	
Transtententorial	1 (1.4)	1 (2.4)	0 (0.0)	
Basale cisterns				n.s. (0.228)
Visible (%)	56 (81.2)	36 (85.7)	20 (74.1)	
Compressed (%)	13 (18.9)	6 (14.3)	7 (25.9)	
Absent (%)	0 (0.0)	0 (0.0)	0 (0.0)	
Ventricles				n.s. (0.522)
Visible (%)	10 (14.5)	7 (16.7)	3 (11.1)	
Compressed (%)	59 (85.5)	35 (83.3)	24 (88.9)	
Absent (%)	0 (0.0)	0 (0.0)	0 (0.0)	
Side of DHC, left, n (%)	37 (53.6)	26 (61.9)	11 (40.7)	n.s. (0.085)

d: largest diameter of the craniectomy, b: perpendicular line with the longest distance from the diameter to the dural flap at the same section as d, DHC: decompressive hemicraniectomy, ICU: intensive care unit IQR: interquartile range RQM: standard reversed question-mark incision, RA: altered posterior question-mark incision, n: number, yr: years.

### Intraoperative complications and outcome

**Figure 3:**
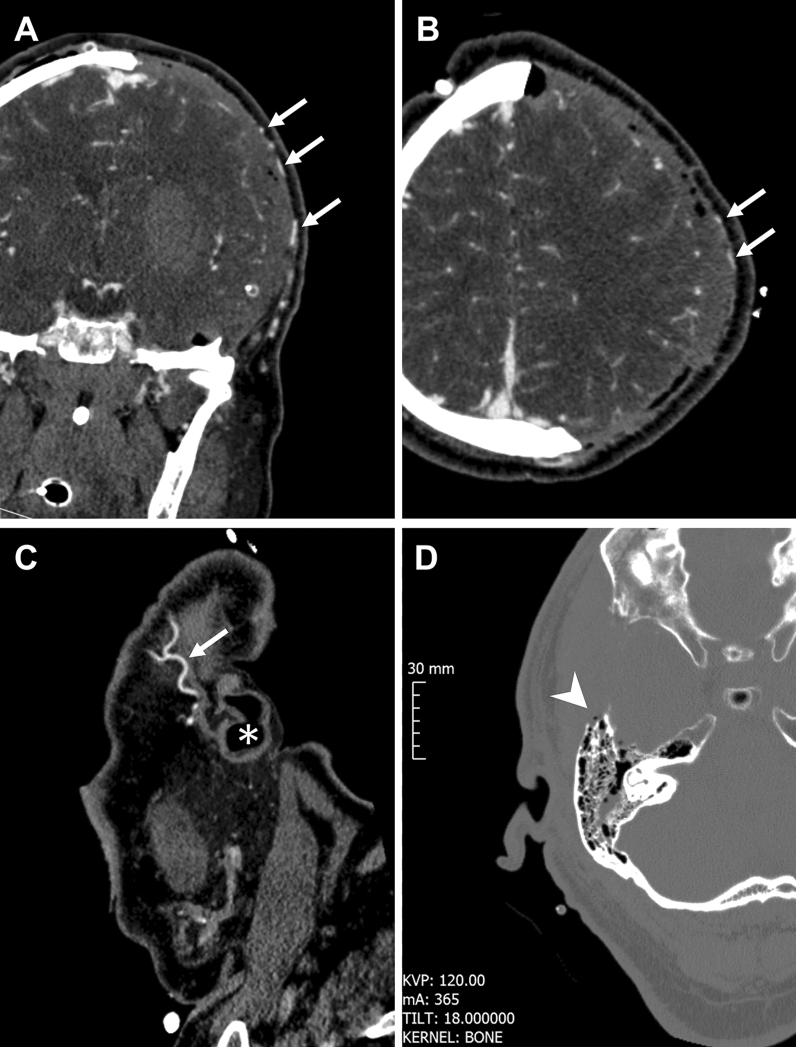
Preservation of the STA and accidental opening of the mastoid air cells. Representative postoperative CT angiography confirming preservation of the STA on (**A**) coronal, (**B**) axial and (**C**) sagittal plane. (**D**) shows breach of the bony air cells of the squamous segment of the temporal bone anterior to the mastoid. Arrows indicate the STA and * marks the external auditory canal. The arrowhead marks the opening of the bony air cells.

There was no difference between the duration of surgery and the median lengths of stay in the ICU. Wound-healing disorders requiring revision surgery after DHC were similar in the modified standard reversed and altered posterior question-mark group (p = 0.344). Further there was no difference between the groups concerning accidental openings of the mastoid cells. All accidental openings of airy cells listed as complications were air cells of the squamous segment of the temporal bone associated with the mastoid (Fig. 3D). There were no associated cerebrospinal fluid fistulae documented in the postoperative period. There was no opening of the transverse sinus and/or an ear injury to report. STA was preserved in all present angiographies in both groups (Fig. 3). Additionally, the data suggested no difference concerning wound infections (p = 0.578) and bone lysis (p = 0.189) after performed cranioplasty between both groups. The outcome of the study population stratified according to the incision type is provided in Table 3.

**Table 3:** Outcome and complications of patients and stratification regarding performed incision technique.

	Total study population (n = 69)	RQM (n = 42)	RA (n = 27)	p-value
Duration of surgery, min, mean $\pm$ SD	121 $\pm$ 44	114 $\pm$ 30	133 $\pm$ 60	n.s. (0.480)
ICU length of stay, days, mean $\pm$ SD	14 $\pm$ 8	15 $\pm$ 8	15 $\pm$ 8	n.s. (0.709)
WHD with need for surgery, n (%)	17 (24.6)	12 (28.6)	5 (18.5)	n.s. (0.344)
Ear injury, n (%)	0 (0.0)	0 (0.0)	0 (0.0)	n.s.
Accidentally opening of the mastoid air cells, n (%)	15 (21.7)	6 (14.3)	9 (33.3)	n.s. (0.061)
Accidentally opening of the transverse/sigmoid sinus, n (%)	0 (0.0)	0 (0.0)	0 (0.0)	n.s.
Postoperative angiography available	n = 46	n = 24	n = 22	
STA preserved, n (%)	46 (100)	24 (100)	22 (100)	n.s.
CP performed	n = 50	n = 34	n = 16	
Time between DHC and CP, days, mean $\pm$ SD	120 $\pm$ 86	112 $\pm$ 70	133 $\pm$ 101	n.s. (0.879)
Reoperation after CP needed, n (%)	5 (11.6)	2 (4.8)	3 (18.8)	n.s. (0.262)
Wound infection after CP, n (%)	4 (9.3)	2 (7.4)	2 (12.5)	n.s. (0.578)
Lytic CP, n (%)	1 (2.3)	0 (0.0)	1 (6.3)	n.s. (0.189)

CP: cranioplasty, DHC: decompressive hemicraniectomy, ICU: intensive care unit IQR: interquartile range RQM: standard reversed question-mark incision, RA: altered posterior question-mark incision, n: number, yr: years, WHD: wound healing disorder.

### Questionnaire regarding personal experience with the altered posterior question-mark incision

Personal experience with the altered posterior question-mark incision was assessed using a 4-point scale (Table 4). Overall, surgeons were highly satisfied with the altered posterior question-mark incision and reported that it was not difficult to reach the temporal base and to sufficiently remove the temporal bone down to the cranial base of the middle cerebral fossa. None of the queried issues were described as unacceptable.

**Table 4:** Experience of the surgeons with the new incision technique (n = 14).

	Excellent	Good, but not optimal	Poor, but acceptable	Unacceptable
Decompression of temporal base, n (%)	11 (78.6)	3 (21.4)	0 (0)	0 (0)
Access to temporal base, n (%)	10 (71.4)	4 (28.6)	0 (0)	0 (0)
Occipital decompression, n (%)	10 (71.4)	4 (28.6)	0 (0)	0 (0)
Control of bleedings, n (%)	13 (92.9)	1 (7.1)	0 (0)	0 (0)
Intraoperative localization of the ear, n (%)	4 (28.6)	7 (50.0)	3 (21.4)	0 (0)
Protection of the ear, n (%)	3 (21.4)	11 (78.6)	0 (0)	0 (0)
Size of craniotomy, n (%)	13 (92.9)	1 (7.1)	0 (0)	0 (0)
Application of wound closure, n (%)	8 (57.1)	5 (35.7)	1 (7.1)	0 (0)

n: number.

## Discussion

The major finding of our study is that the altered posterior question-mark incision, which starts behind the ear at the mastoid notch, allows for an equally effective decompression of the temporal base and for the same size of the craniectomy. The rate of additional intraoperative complications appeared to be low. However, the STA could be equally preserved in both groups and there was no difference in the rate of postoperative wound-healing complications.

Wound-healing disorders belong to the most frequently encountered complications after DHC and in the literature, the rate of wound failure varies between 3 and 40%^10^. The main reason for the increased risk is supposed to be due to the large skin and bone flap and the increased risk of injuring the STA during the widely used standard reversed question-mark incision, leading to reduced perfusion and ischemia of the large skin flap^8^. The recently proposed altered posterior question-mark incision starts behind the ear and posterior of the base of the mastoid process and is supposed to better preserve the STA during incision^11,13^. However, beginning behind the ear, it is unclear if this incision allows for the same size of bone flap as well as for a sufficient exploration and decompression of the temporal base compared to the standard reversed question-mark incision. With the standard reversed question-mark incision, the inferior line is usually extended below the level of the zygomatic arch and the scalp is then reflected along with the temporalis muscle anteroinferiorly^19^. This allows for a maximum retraction of the combined myocutaneous flap down to the cranial base, in order to explore and adequately remove the temporal bone. An adequate bony decompression over the lateral temporal lobe down to the middle cerebral fossa is essential for an optimized decompression of the brainstem. Previous studies demonstrated a correlation of the state of the mesencephalic cisterns with the distance of the lower border of the craniectomy to the temporal base^21^ and it is well established, that abnormal cisterns increase the risk of critically high ICP and worse outcome after severe brain injury^22,23^. A sufficient diameter of the craniectomy of at least 15 cm also demonstrated to reduce the risk of external brain herniation and improved survival and favorable functional outcome after DHC^20,24,25^. Using the altered posterior question-mark incision starting behind the ear, however, the skin flap might be hindered by the attachment of the external auditory canal, making it more difficult to sufficiently reflect the myocutaneous flap anterioinferiorly in order to reach the lesser wing of the sphenoid and the squamous segment of the temporal bone^11^. It might also be more difficult to extend the craniectomy to the frontal squamous bone in order to achieve the necessary diameter of the bone flap. Therefore, in this study we evaluated if the size of the removed bone flap and the decompression of the temporal base differs between our modified standard reversed and the altered posterior question-mark incision. The modified standard reversed question-mark incision that we used to perform at our institution, starts slightly before the ear and superior to the pinna but is not extended down to the zygomatic arch. This modified incision allows for an equally sufficient retraction of the myocutaneous flap compared with the standard incision starting at the zygomatic arch, but is supposed to avoid crossing the STA.

Intraoperatively, we did not experience more difficulty to reach the temporal base and to remove the temporal bone with a rongeur using the altered posterior question-mark incision. This was confirmed by means of postoperative CT scans which demonstrated an equally effective decompression of the temporal base with no difference in the distance of the lower margin of the decompression down to the floor of the middle cerebral fossa. The median distance between the lower border of the craniectomy and the temporal cranial base in both groups was equal and in accordance with results previously reported on the standard reversed question-mark incision with extension down to the zygomatic arch^21,26^. The similar decompression of the temporal base in our study was also reflected in the state of the basal cisterns, which were mostly open in both groups. Further, there was no difference in size of the removed bone flap. Therefore, the altered posterior question-mark incision allows for the same size of craniectomy and for an equally effective decompression of the temporal base. But there are other risks suggested to be associate with the altered incision. If the incision is extended below the mastoid process, there is a risk of accidentally opening the mastoid air cells and/or the transverse/sigmoid sinus^12,15^. Veldeman et al. even described a case during which the external auditory canal was opened^16^. The additional risk of intraoperative complications using the posterior incision was low in our cohort and might be easily avoided if the craniotomy stays superior of the transverse sinus and if the scalp flap is not retracted too forcefully. Careful downward retraction of the myocutaneous flap also prevents injuring the ear. In most cases the mastoid notch and the nuchal line can be easily identified and used as landmarks for the localization of the sinuses and the transverse-sigmoid junction before craniotomy. In our study, we did not experience any injury of the ear and did not encounter a significant an increased rate of accidental opening of the mastoid air cells or the transvers/sigmoid sinus. However, our data suggest that there may be a higher risk for accidental openings of the mastoid air cells using the altered posterior question-mark incision. Therefore, the authors recommend institutions switching to the altered posterior question-mark incision to be aware of this potential specific pitfall. Most importantly, there was no difference between both groups´ rate of intraoperatively preserved and open STA on postoperative angiography imaging. This can be primarily attributed to the fact, that the modified standard reverse question mark incision in our study starts superior to the pinna without extension in front of the ear and down to the zygomatic arc, allowing for an equally effective preservation of the STA. However, the modified standard reversed question-mark incision might injure the parietal branch, which is more likely to be preserved with the altered posterior incision and might not be reliably identified on the postoperative angiographies.

Still, we did not find a significant reduction in infections or wound-healing disorders requiring revision surgery using the altered posterior question-mark incision. However, the present study was not designed to detect differences in postoperative wound-healing disorders and the data shows a trend towards an advantage of the altered posterior question-mark incision regarding wound healing. The results are also in accordance with the findings by Veldeman et al., who demonstrated a significantly reduced rate of infection and wound-healing disorders after cranioplasty but not after primary DHC^11^. This suggests, that reduced scalp flap perfusion and the role of the incision type is more relevant after cranioplasty than it is right after DHC. Regarding the early postoperative phase after DHC, several other factors have been suggested to influence infection rate and wound-healing disorders. Recently, we demonstrated an increased rate of postoperative subgaleal CSF accumulations on the ipsilateral side after DHC, significantly impacting wound-healing disorders and the rate of surgical revisions. Subgaleal CSF accumulations cause additional tension to the wound and can be reduced using early postoperative lumbar CSF drainage^27^. In this study, only a few patients had a postoperative lumbar drainage in both groups, minimizing this bias. Nevertheless, the novel posterior question-mark incision allows for an equally effective decompression without increased risk of intraoperative complications and a previous study demonstrated a reduced rate of wound-healing complications after cranioplasty, we changed our clinical practice in DCH by using the novel posterior question-mark incision starting behind the ear. However, bigger studies are needed to confirm the benefit of the new incision type on postoperative wound-healing complications and to demonstrate the superiority of the altered posterior question-mark incision for DHC.

## Limitations

Due to the retrospective nature, our single-center study is subject to various well-known limitations. First, the small patient number and heterogenous population might fail to detect significant differences in the rate of intraoperative complications and postoperative wound-healing disorders. Further, long-term wound infections and healing complications might not have been reported due to the variably and missing active follow-up after cranioplasty. However, patients are seldom referred to another center if postoperative complications occur. Therefore, it is unlikely that we missed a relevant number of patients with postoperative complications or wound-healing disorders. At least, the follow-up period was too short to detect or report all aseptic bone resorptions after DHC as previous study demonstrated that aseptic bone resorption occurs after a median time of 15 months after primary DHC^28^. Furthermore, the authors cannot exclude that the high rate of preserved STAs in our study with the altered posterior question-mark incision may be attributed to our expertise as a vascular neurosurgical center. The observed rate may potentially differ in other institutions.

## Conclusion

Sufficient decompression substantially influences outcome after DHC and the altered posterior question-mark incision for DHC demonstrated to allow for an equally effective decompression of the temporal base, while the STA could be preserved in all cases. Further, the rate of intraoperative complications was low and therefore, the altered posterior question-mark incision demonstrates to be a safe and effective alternative to the standard reversed question-mark incision. However, previously described reduction in wound-healing complications and cranioplasty failures needs to be confirmed in prospective studies to demonstrate the superiority of the altered posterior question-mark incision.

## Data Availability

The datasets generated during and/or analysed during the current study are available from the corresponding author on reasonable request.
